# Positioning Velocity Matters in Central Paroxysmal Positional Vertigo: Implication for the Mechanism

**DOI:** 10.3389/fneur.2020.591602

**Published:** 2020-10-22

**Authors:** Xia Ling, Hyo-Jung Kim, Jong-Hee Lee, Jeong-Yoon Choi, Xu Yang, Ji-Soo Kim

**Affiliations:** ^1^Department of Medicine, Seoul National University College of Medicine, Seoul, South Korea; ^2^Research Administration Team, Seoul National University Bundang Hospital, Seongnam, South Korea; ^3^Dizziness Center, Seoul National University Bundang Hospital, Seongnam, South Korea; ^4^Department of Neurology, Seoul National University College of Medicine, Seoul, South Korea; ^5^Department of Neurology, Aerospace Center Hospital, Peking University Aerospace School of Clinical Medicine, Beijing, China

**Keywords:** vertigo, nystagmus, central positional nystagmus, downbeat nystagmus, mechanism

## Abstract

**Objectives:** To elucidate the mechanism of paroxysmal central positional nystagmus (CPN) by determining the effects of head rotation velocity on the intensity of paroxysmal downbeat nystagmus induced during straight head hanging (SHH).

**Methods:** We recruited 21 patients with paroxysmal downbeat CPN induced during SHH at the Dizziness Center of Seoul National University Bundang Hospital from September 2018 to July 2019. Twenty-one patients had manual SHH at two different lying velocities, the fast (routine) and slow, and they also underwent SHH at different rotation velocities of 10, 20, 30, and 40 °/s using a motorized rotation chair. Induced nystagmus was recorded using video-oculography and the maximum slow phase velocity (SPV) and time constant (TC) of the induced paroxysmal nystagmus were analyzed.

**Results:** During manual SHH, paroxysmal downbeat nystagmus was invariably induced during routine SHH (fast lying down), but absent or minimal during slow positioning. During motorized SHH, the median of maximum intensity of downbeat nystagmus increased from 7.6 °/s (0–16.9) to 14.0 °/s (0–32.5), 16.5 °/s (0–44.6), and 19.1 °/s (0–55.2) as the rotation velocity increased from 10 to 20, 30, and 40°/s (*P* < 0.001, *P* < 0.001, *P* = 0.004; linear mixed models). In contrast, the TCs of paroxysmal downbeat CPN remained unchanged (*P* = 0.558, *P* = 0.881, *P* = 0.384, linear mixed models).

**Conclusions:** The dependence of nystagmus intensity on head rotation velocity supports a disinhibited and exaggerated inhibitory rebound of the canal signals as the mechanism of paroxysmal CPN.

## Introduction

Either paroxysmal or persistent form of positional vertigo and nystagmus may occur in central as well as peripheral vestibular disorders (1). In peripheral disorders, paroxysmal vertigo and nystagmus are mostly explained by migration of the otolithic debris dislodged from the utricular macule into the semicircular canals or by alteration in the specific gravity of the cupula relative to endolymph (2). Central positional nystagmus (CPN) mostly takes the form of apogeotropic (3) or geotropic (4) nystagmus when the head is turned to either side while supine, or downbeat nystagmus after lying down (5).

Persistent CPN has been explained by erroneous neural processing within the velocity-storage circuit located in the brainstem and cerebellum that functions in estimating the angular head velocity, gravity direction, and inertial acceleration (3). Paroxysmal CPN is also featured by transient geotropic or apogeotropic nystagmus after head-turning to either side while supine, and upbeat or downbeat nystagmus after lying down, straight head hanging (SHH) or sitting up (6). Of those, paroxysmal downbeat nystagmus while lying down or SHH is most common (7). The mechanism of paroxysmal CPN requires further exploration. In our previous study, the paroxysmal form of CPN mostly occurred in the planes of semicircular canals inhibited during the positioning, and showed the features suggestive of a semicircular canal origin regarding the latency, duration, and direction of nystagmus (7). Based on those findings, paroxysmal CPN was attributed to disinhibition and enhanced responses of the secondary vestibular neurons during the positioning due to lesions involving the nodulus and uvula (7). In this instance, increment of the post-rotatory signals is invoked to generate paroxysmal CPN (7). For example, paroxysmal downbeat nystagmus observed during lying down or SHH is explained by transient over-activation of the anterior and/ or horizontal canals on both sides (7). According to this explanation, the intensity of paroxysmal CPN would depend on stimulation intensity of the semicircular canals during the positioning, and thus lying velocity (acceleration) in paroxysmal downbeat CPN.

This study aimed to determine the effect of lying (head rotation) velocity (acceleration) on the intensity of paroxysmal downbeat CPN induced during SHH by adopting a stepwise increase in the rotation velocity. We hypothesized that the intensity of induced nystagmus would increase in proportion to the lying velocity.

## Materials and Methods

### Patients

We recruited 27 patients with paroxysmal downbeat CPN at the Dizziness Center of Seoul National University Bundang Hospital from September 2018 to July 2019. All experiments followed the tenets of the Declaration of Helsinki and this study was approved by the Institutional Review Board of Seoul National University Bundang Hospital (IRB No. B-1908/558-103).

All patients underwent detailed neuro-otologic evaluation by the senior author (J.S.K). The diagnosis of paroxysmal downbeat CPN was based on (1) paroxysmal downbeat nystagmus (<1 min) induced during SHH, (2) presence of other symptoms and signs indicative of brainstem or cerebellar dysfunction, or brainstem or cerebellar lesions documented on MRIs, and (3) no resolution of paroxysmal downbeat nystagmus with repeated canalith repositioning maneuvers for benign paroxysmal positional vertigo involving the anterior semicircular canals. Of the 27 patients, 21 (12 men, mean age ± *SD* = 54.4 ± 14.9 years) were finally included for analyses after excluding six patients due to (1) a duration of the paroxysmal nystagmus <5 s (*n* = 1), (2) concomitant periodic alternating nystagmus that interfered accurate analyses of downbeat nystagmus (*n* = 1), (3) an inconsistency; direction-changing or alternating with horizontal nystagmus (intermittent, *n* = 2), and (4) incomplete evaluation (*n* = 2).

### Oculography

Eye movements were recorded binocularly using 3-dimensional video-oculography (VOG, SLVNG®, SLMED, Seoul, South Korea). Spontaneous nystagmus was recorded both with and without visual fixation in the sitting position. Gaze-evoked nystagmus, horizontal smooth pursuit, horizontal saccades, and horizontal head-shaking nystagmus were also evaluated.

### Positioning Maneuvers

**Figure 1:**
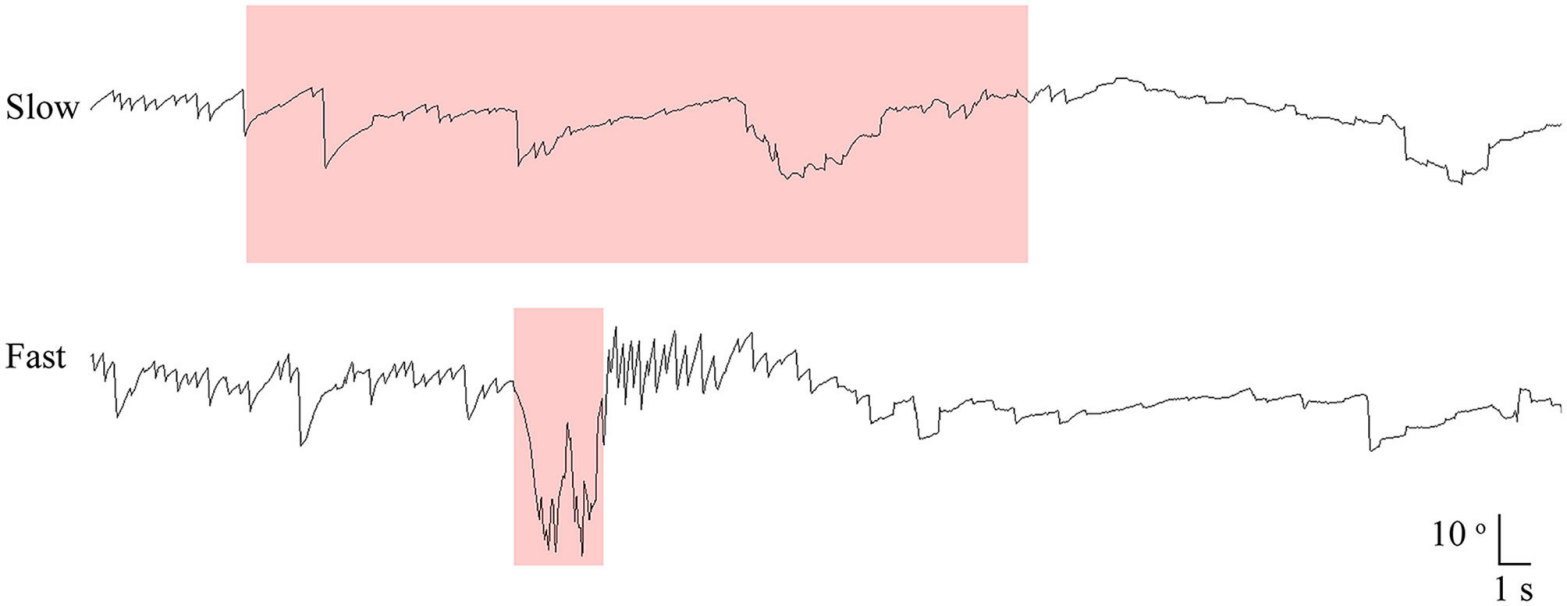
Recording of paroxysmal downbeat nystagmus during manual straight head hanging in patient 21. With slow positioning, no discernable nystagmus was induced, and the pre-existing spontaneous downbeat nystagmus was even suppressed. During fast positioning, in contrast, paroxysmal downbeat nystagmus was induced for several seconds. Only the vertical eye motion is presented and the shaded areas in pink indicate the period of positioning. Upward deflection denotes upward eye motion in each recording.

All patients had manual SHH at both fast (routine) and slow lying velocities. During the fast SHH, patients were laid from the sitting position onto the lying down position with the head extended about 30° below the table. During the fast SHH, this positioning was performed at a routine velocity and was completed usually within 3 s (2–4 s). Thus, the mean head rotation velocity was about 40°/s during the fast SHH test. During the slow SHH, the patients assumed the same position over about 35 s (from 25 to 51 s) with an assistance by the examiner. Thus, the mean head rotation velocity was ~3–4 °/s during the slow SHH (Figure 1, Supplementary Video 1).

**Figure 2:**
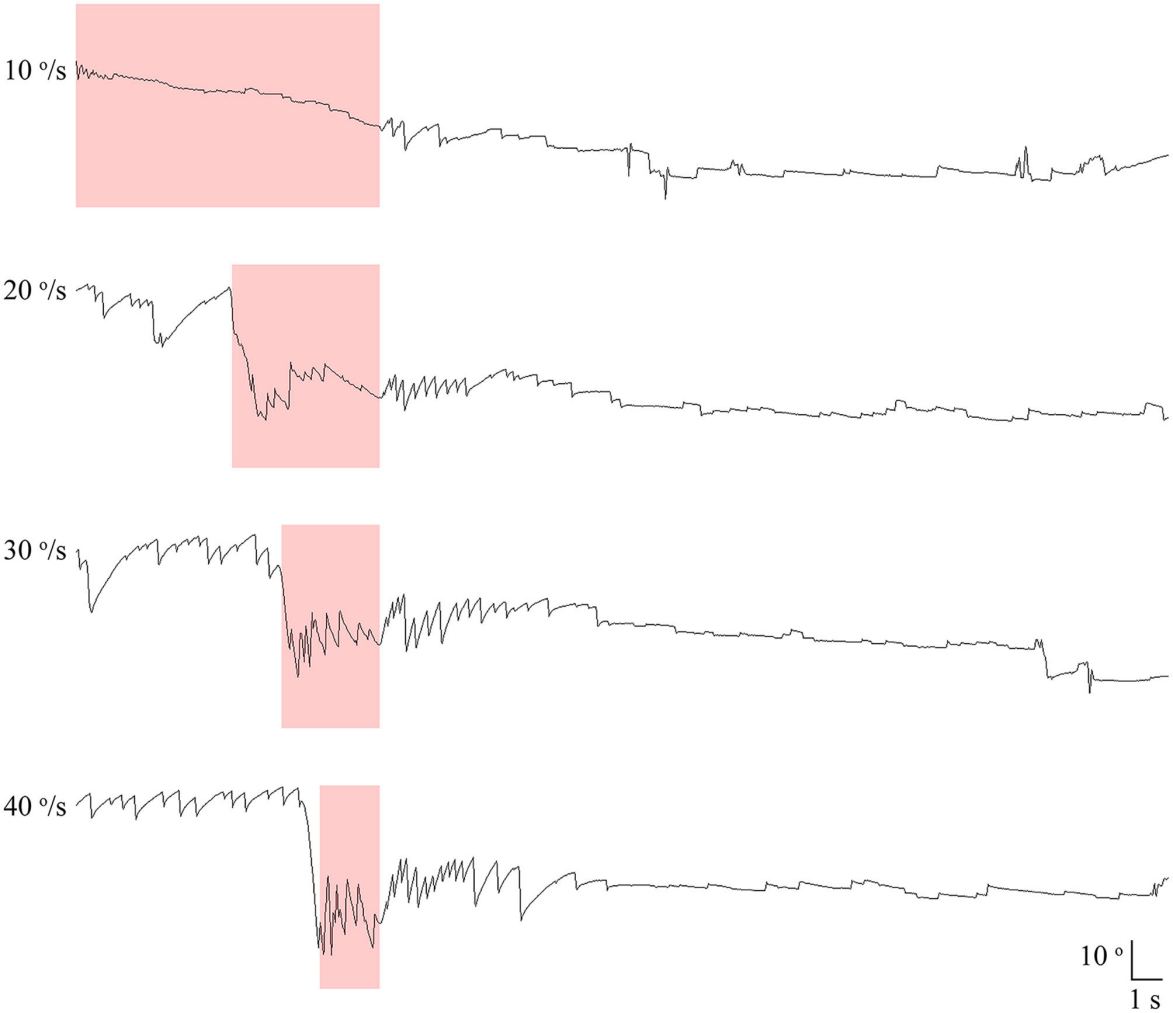
Recording of paroxysmal downbeat nystagmus induced during motorized rotation chair test at four different rotation velocities in patient 21. The intensity of induced downbeat nystagmus increased as the rotation velocity was escalated from 10 to 40°/s. The paroxysmal downbeat nystagmus was followed by a small upbeat nystagmus. Only the vertical eye motion is presented and the shaded areas in pink indicate the period of positioning. Upward deflection denotes upward eye motion in each recording.

All patients also underwent SHH at different rotation velocities of 10, 20, 30, and 40 °/s using a 3-dimensional motorized rotation chair (SLRVT®, SLMED, Seoul, South Korea, Figure 2, Supplementary Video 2). During this maneuver, the patients were tilted backward 100° from the sitting position without extending the neck at each rotation velocity.

In each testing condition, the SHH position was maintained until the positional nystagmus disappeared or at least for 1 min. The positional nystagmus was recorded at a sampling rate of 120 Hz without visual fixation in darkness using video-oculography (VOG, SLVNG®, SLMED, Seoul, South Korea).

### Analyses of Nystagmus

Digitized eye position data were analyzed with MATLAB software (version R2019b, The MathWorks, Inc., MA, USA). For each paroxysmal CPN, the intensity (maximum slow phase velocity, SPV) and time constant (TC) were calculated. When persistent CPN was combined with the paroxysmal one, we calculated the maximum SPV and TC of paroxysmal CPN after subtracting the persistent component from the velocity profile of induced positional nystagmus.

### Statistical Analyses

Statistical analyses were performed using SPSS software (version 20.0, IBM SPSS Statistics, N.Y., USA). Continuous variables were expressed as a mean ± *SD* for parametric values or as a median (range) for non-parametric ones. Counting variables were expressed as a percentage. Normality of the data was determined using the Shapiro-Wilk test. Linear mixed model analysis was used to determine any difference in the maximum SPV and TC of paroxysmal downbeat CPN among the test conditions that had adopted different rotation velocities using a motorized rotation chair. The level of statistical significance for the linear mixed model analyses was corrected using the Bonferroni method, and the corrected level of significance was set at 0.0083 (0.05/6) since the comparisons were performed six times in each group. The Wilcoxon signed ranks test were used to determine any difference in the maximum SPVs of paroxysmal downbeat CPN induced during the fast and slow SHH and maximum SPVs of paroxysmal downbeat CPN induced during the fast SHH and motorized positioning test at a rotation velocity of 40.0°/s, and the paired *t*-test was used to determine any difference in the TCs of paroxysmal downbeat CPN induced during the fast SHH and motorized positioning test at a rotation velocity of 40.0 °/s. The significance threshold was set at a 2-sided *P* < 0.05.

## Results

### Clinical Characteristics

Underlying disorders included spinocerebellar ataxia (*n* = 14), episodic ataxia (*n* = 4), Chiari malformation (*n* = 1), and no etiology was identified in the remaining two patients.

During visual fixation, eight patients (38.1%) showed spontaneous nystagmus that was pure downbeat in seven, and mixed horizontal and downbeat in the remaining one. The SPV of downbeat nystagmus ranged from 0.7 to 8.2°/s (median = 2.0). Without visual fixation in darkness, 15 patients (71.4%) showed spontaneous nystagmus that was pure downbeat in eight, mixed horizontal-downbeat in five, pure horizontal in one, and mixed horizontal-upbeat in the remaining one. The SPV of downbeat nystagmus ranged from 1.1 to 8.7°/s (median = 3.1) without visual fixation in darkness. After horizontal head-shaking, 18 patients (85.7%) showed nystagmus that was mixed horizontal-downbeat in 11, pure downbeat in five, and pure horizontal in two. Gaze-evoked nystagmus was found in 14 patients (66.7%), and eight of them also showed rebound nystagmus. Horizontal smooth pursuit was impaired in 14 patients (66.7%). Horizontal saccades were abnormal in 11 patients (52.4%); hypermetric in six, hypometric in four, and slow in one (Table 1).

**Table 1:** Findings in patients with paroxysmal downbeat central positional nystagmus.

**Pt**	**Etiology**	**Sex/Age**	**SN fix**	**SN non-fix**	**GEN**	**HSN**	**Manual**				**Rotatory chair**							
							**Max SPV**		**TC**		**Max SPV**				**TC**			
							**Slow**	**Fast**	**Slow**	**Fast**	**10**°** /s**	**20**°** /s**	**30**°** /s**	**40**°** /s**	**10**°** /s**	**20**°** /s**	**30**°** /s**	**40**°** /s**
1	SCA 6	M/56	-	D	-	R+D	0.0	14.5	-	3.4	8.7	14.6	14.7	16.5	7.1	7.8	6.3	4.5
2	ACM	F/66	D	R+D	+(REB)	R → L+D	0.0	30.0	-	4.3	6.7	11.2	15.8	21.9	7.7	4.1	4.3	4.3
3	SCA	F/45	D	R+D	+(REB)	R+D	0.0	16.0	-	7.4	11.4	16.4	20.3	18.2	2.9	4.0	3.8	4.1
4	CA	F/70	-	L	+(REB)	-	10.4	25.7	1.3	1.4	8.0	16.5	22.1	23.3	2.3	2.8	1.5	1.1
5	CA	F/72	-	-	+(REB)	R → L	12.3	23.1	3.7	6.2	9.2	12.1	12.1	13.5	3.0	4.9	4.3	5.7
6	SCA 1	M/62	-	D	-	R → L+D	0.0	16.2	-	2.0	6.4	11.3	14.2	18.5	2.9	1.9	1.5	1.5
7	EA2	F/22	D	R+D	-	L → R+D	0.0	19.8	-	2.0	0.0	6.5	17.4	24.1	-	3.0	2.6	2.4
8	CA	M/48	-	-	+	D	0.0	11.6	-	5.3	15.7	23.8	22.8	16.3	4.0	3.2	4.6	5.9
9	CA	F/43	-	D	-	R+D	0.0	19.5	-	4.5	8.5	14.0	16.5	19.5	2.6	4.0	4.2	3.7
10	CA	M/72	-	L+D	-	L+D	0.0	26.7	-	3.5	5.5	8.0	8.4	6.7	3.1	3.1	3.2	3.8
11	CA	M/47	-	U+OF	+	-	0.0	35.9	-	2.7	16.8	17.0	19.4	22.4	1.0	2.8	2.7	2.7
12	CVUE	M/67	D	D	+	D	0.0	19.4	-	3.7	0.0	13.9	16.3	19.1	-	1.4	3.4	2.3
13	EA2	M/16	D	D	+(REB)	L+D	0.0	26.1	-	3.5	7.6	8.3	10.4	13.8	3.6	3.3	5.7	2.0
14	EA	M/48	-	D	-	D	0.0	4.6	-	6.8	0.0	0.0	3.8	4.1	-	-	6.5	6.8
15	CA	F/55	-	-	+(REB)	-	0.0	23.6	-	1.1	0.0	23.2	27.4	35.1	-	0.8	1.4	1.3
16	SCA	M/66	D	D	+	R → L+D	0.0	22.2	-	4.7	10.8	24.7	25.6	28.8	2.8	3.6	4.4	4.2
17	SCA	F/48	-	-	+(REB)	D	0.0	17.5	-	2.7	13.8	21.2	26.0	30.2	2.3	2.4	2.1	1.9
18	OPCA	F/56	-	-	-	D	0.0	9.3	-	6.5	6.5	9.5	9.7	9.8	3.7	4.2	4.1	4.4
19	EA	M/63	-	-	+	R+D	0.0	6.8	-	3.3	0	0	0	0	-	-	-	-
20	CVUE	M/55	D	D	+	R	0.0	56.7	-	1.8	0	20.9	21.7	34.1	-	1.8	1.8	1.9
21	SCA	M/65	D+SWJ	D+SO	+(REB)	L → R+D	0.0	65.6	-	2.7	16.9	32.5	44.6	55.2	1.4	2.7	2.9	2.7

*ACM, Arnold-Chiari malformation; APAN, aperiodic alternating nystagmus; CA, cerebellar ataxia; CVUE, central vertigo with unknown etiology; D, downbeat nystagmus; EA, episodic ataxia; F, female; GEN, gaze-evoked nystagmus; HSN, head shaking nystagmus; L, left beating nystagmus; M, male; Max, maximum; MSO, macro saccadic oscillation; OF, ocular flutter; OPCA, olivopontocerebellar atrophy; Pt, patient; R, right beating nystagmus; REB, rebound; SCA, spinocerebellar ataxia; SPV, slow phase velocity; SN, spontaneous nystagmus; SN fix, spontaneous nystagmus with visual fixation; SN non-fix, spontaneous nystagmus without visual fixation; SO, saccadic oscillation; SWJ, square wave jerk; TC, time constant; +, present or plus; −, normal or absent; →, change to*.

### Manual SHH

**Figure 3:**
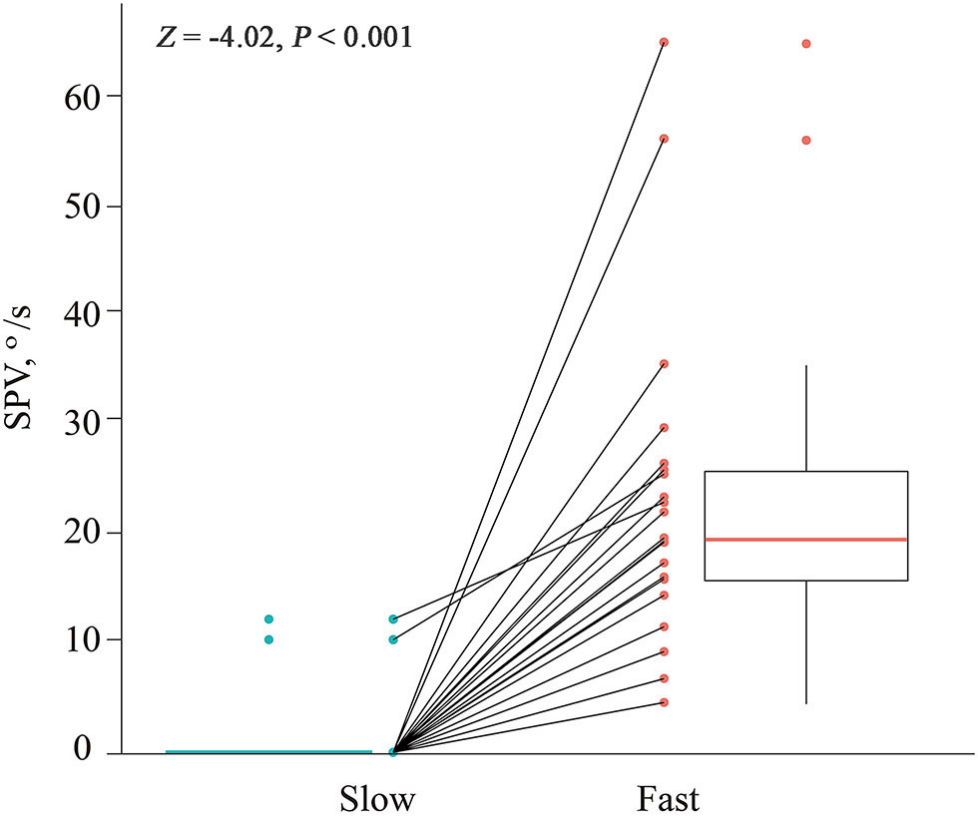
Comparison of the maximum slow phase velocity (SPV) of paroxysmal downbeat nystagmus induced during slow and fast manual straight head hanging tests in each patient. During slow positioning, only two patients showed paroxysmal downbeat CPN, which became larger during fast positioning. In contrast, paroxysmal downbeat nystagmus was observed in all patients after fast positioning.

All 21 patients showed paroxysmal downbeat CPN during the routine (fast) positioning, and only two of them showed downbeat nystagmus during the slow SHH (Table 1). During the routine SHH, the downbeat CPN was pure paroxysmal in 16 and mixed paroxysmal and persistent in five patients. The paroxysmal downbeat CPN showed a peak initially in 19 patients (90.5%) and reached its peak within 2.5 s in the remaining two. After then, the nystagmus decreased exponentially. The maximum SPV of positional downbeat nystagmus ranged from 4.6 to 65.6°/s (median = 19.8, mean ± *SD* = 23.4 ± 14.7), and the TC ranged from 1.1 to 7.4 s (median = 3.5, mean ± *SD* = 3.8 ± 1.8) during the fast SHH. During the slow SHH, the maximum SPVs for the two patients with discernable positional downbeat nystagmus were 10.4 and 12.3°/s with the TCs at 1.3 and 3.7 s. Thus, there was a significant rise in the maximum SPV of paroxysmal downbeat CPN as the lying velocity increased (*Z* = −4.02, Wilcoxon signed ranks test, *P* < 0.001 (Table 1, Figure 3).

### Motorized Rotation Chair Test

**Figure 4:**
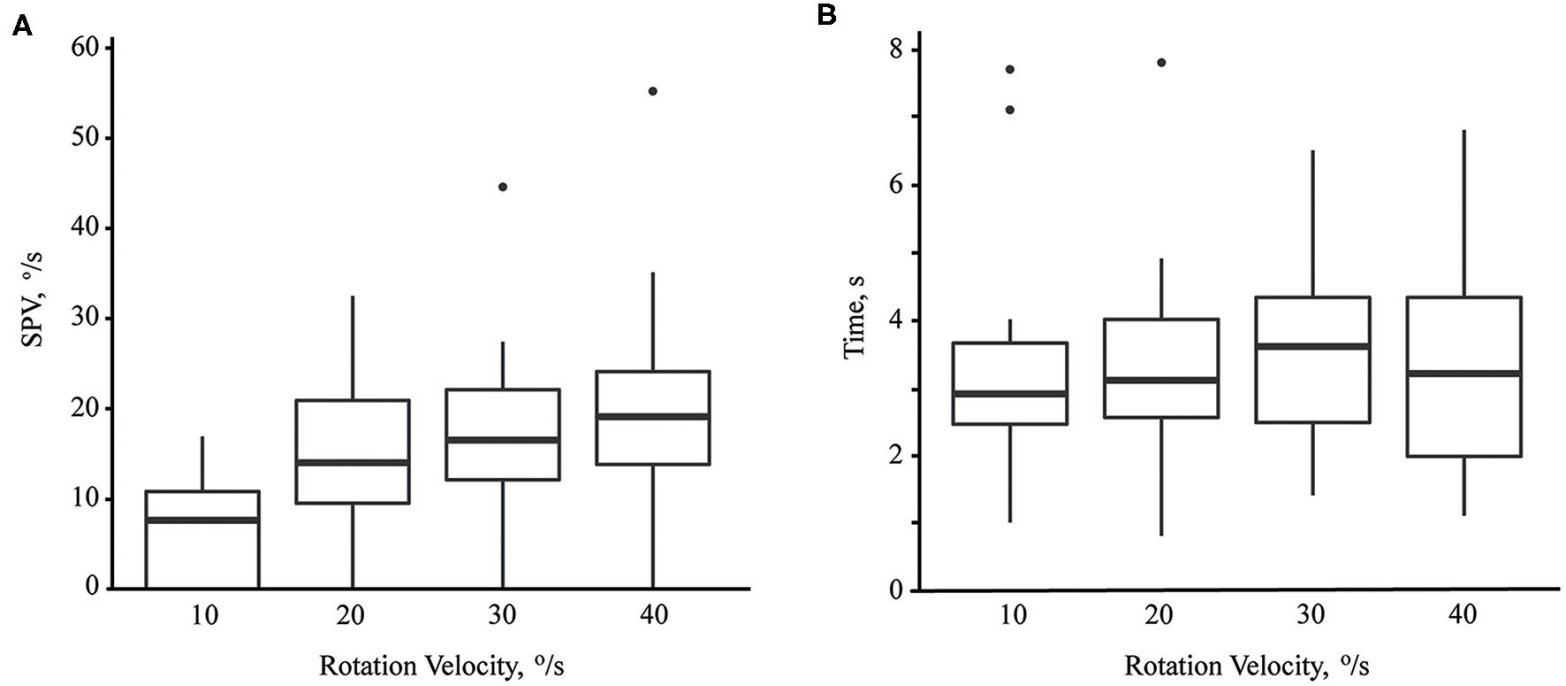
The maximum slow phase velocity (SPV, **A**) and time constant **(B)** of paroxysmal downbeat nystagmus induced during motorized rotation chair test at four different rotation velocities. As the rotation velocity was escalated from 10 to 40°/s, the maximum slow phase velocity of induced downbeat nystagmus increased while the time constant remained unchanged.

The median of maximum SPV of paroxysmal downbeat CPN was 7.6 °/s (range: 0–16.9; mean ± *SD* = 7.2 ± 5.7) at the rotation velocity of 10°/s, which increased to 14.0 °/s (range: 0–32.5; mean ± *SD* =14.6 ± 8.1) at 20°/s, 16.5°/s (range: 0–44.6; mean ± *SD* = 17.6 ± 9.5) at 30°/s, and 19.1°/s (range: 0–55.2; mean ± *SD* = 20.5 ± 12.1) at 40°/s. Thus, there was a significant rise in the maximum SPV of paroxysmal downbeat CPN as the lying velocity increased [(95% CI, −18.42 to −8.19), *P* < 0.001; (95% CI, −8.95 to −3.00), *P* < 0.001; (95% CI, −4.84 to −1.06), *P* = 0.004; Linear mixed model; Figure 4A]. In contrast, the TCs of paroxysmal downbeat nystagmus remained at about 3.5 s without a difference among the four testing conditions [(95% CI, −1.12 to 0.62), *P* = 0.558; (95% CI, −0.51 to 0.60), *P* = 0.881; (95% CI, −0.29 to 0.70), *P* = 0.384, Linear mixed model, Figure 4B].

Six patients showed both paroxysmal and persistent components of positional nystagmus, and the persistent nystagmus was upbeat (0.2–3.1°/s) in five and downbeat (3.1°/s) in one.

### Comparison of Manual and Motorized SHH

We compared the maximum SPVs and TCs of the paroxysmal downbeat nystagmus between the manual fast SHH and motorized SHH test at the rotation velocity of 40 °/s since the head rotation velocities were similar for those testing conditions, and found that the maximum SPVs [19.1 (0–55.2) vs. 19.8 (4.6–65.6) °/s, *Z* = −1.06, Wilcoxon signed ranks test, *P* = 0.29] and TCs [3.8 ± 1.9 vs. 3.4 ± 1.6 s, (95% CI, −0.02–0.92), paired *t*-test, *P* = 0.06] of the paroxysmal downbeat CPN induced during both tests were similar.

## Discussion

We found that the intensity of paroxysmal downbeat CPN during SHH depends on the rotation velocity (acceleration) of the head during the positioning while the TC was not affected by the rotation velocity. Furthermore, the intensity of positional downbeat nystagmus increases in proportion to the positioning velocity during the motorized rotatory chair tests.

In this study, most patients showed diffuse cerebellar dysfunction in association with the hereditary or acquired forms of ataxia. However, the associated ocular motor findings of spontaneous downbeat nystagmus, gaze-evoked nystagmus, perverted head-shaking nystagmus, and impaired smooth pursuit all indicate dysfunction of the vestibulocerebellum (8–10). In our previous studies, circumscribed lesions responsible for CPN, either the paroxysmal or persistent form, also were mostly overlapped in the nodulus and uvula (7).

In this study, the intensity of paroxysmal downbeat CPN induced during SHH depended on the positioning velocity during either manual SHH or the whole-body rotation using a motorized chair. Indeed, most patients showed no or minimal downbeat nystagmus during SHH when the positioning was performed at a very slow speed. This was also observed in a previous study on spontaneous downbeat nystagmus even though the detailed findings or mechanisms were not explored (11). The TCs of paroxysmal downbeat CPN were mostly around 3.5 s with some variations among the patients and different testing conditions, which are similar to the TCs of the vertical semicircular canals (12–14). These characteristics of paroxysmal downbeat nystagmus observed in this study are consistent with those found in our previous study on paroxysmal CPN regarding its maximum at onset, short duration with a TC around 4 s, and alignment of the nystagmus direction with the vector sum of the rotational axes of the semicircular canals that are normally inhibited during the positioning (7). Based on these findings, we previously proposed that paroxysmal CPN is generated by disinhibition and enhanced responses of the secondary vestibular neurons during positioning due to cerebellar dysfunction (7). The similar TCs regardless of different nystagmus intensity in each testing condition are consistent with the findings observed in previous studies on the attenuation of nystagmus but constant TC during rotations having adopted a stepwise increase in the head rotation velocity (15, 16).

The first-order neurons from the horizontal semicircular canal have a resting discharge and the firing rate increases during head rotation that induces an ampullopetal flow of the endolymph and decreases during head motion that produces an ampullofugal flow of the endolymph. The reverse holds for the vertical semicircular canals (17, 18). Within a specific dynamic range, this increase or decrease of action potentials is directly proportional to the magnitude of head acceleration or deceleration (17). The primary vestibular afferents are classified into the regular and irregular according to their discharge properties (17). The irregular afferents are responsible for the adaptation responses and velocity-storage mechanism. The adaptation responses include a rapid decline in the discharge with a rebound when the discharge goes below the resting rate and a following gradual return to the resting level (post-acceleratory secondary phenomenon) (17). We postulate that the lesions involving the vestibulocerebellum disinhibit the irregular afferents and result in exaggerated post-acceleratory secondary phenomenon (7). One of the functions of the velocity-storage mechanism is to estimate the gravitational direction by integrating the rotation cues from the semicircular signals. Since there is a rotation feedback loop inside the velocity-storage mechanism, which acts to adjust the estimated gravitational direction to the real gravitational direction (7), vestibulocerebellar lesions and resultant disinhibition of the irregular afferents would lead to an exaggerated post-acceleratory secondary response and a difference between the estimated and real gravitational directions. This difference would generate the post-rotatory nystagmus (7). During SHH, the irregular vestibular fibers from both posterior semicircular canals are mostly activated and this activation is followed by transient inhibitory rebound (17). When this secondary inhibition becomes prominent due to dysfunction of the vestibulocerebellum, the exaggerated inhibitory signals conveyed to the vestibular storage mechanism may generate nystagmus through the rotational feedback mechanism (7). In this scenario, the degree of post-acceleratory depression would depend on the strength of head acceleration during positioning, which forms the basis of this study. Indeed, the intensity of paroxysmal downbeat CPN induced during SHH depended on the positioning head velocity.

Several studies showed that transient compression of the vertebral arteries during neck rotation or extension may reduce the blood supply to the peripheral or the central parts of the vestibular system and give rise to paroxysmal downbeat nystagmus along with vertigo and tinnitus (19–21). The presumed mechanism was excitation of unilateral or bilateral anterior semicircular canals (19–21). Thus, paroxysmal downbeat nystagmus may be induced by head extension during SHH. However, in our study, only two patients (9.5%) showed paroxysmal downbeat CPN during the manual slow SHH test, and all patients showed paroxysmal downbeat CPN during the SHH using a rotation chair, which did not allow any neck motion during the positioning. Thus, the role of head extension, if any, in generating paroxysmal downbeat nystagmus could have been minimal in our study.

Four patients showed an evolution of initial positional downbeat into upbeat nystagmus over 4–15 s. This reversal in the direction of induced nystagmus is observed in various conditions including benign paroxysmal positional vertigo (22–24), head-shaking nystagmus (25), caloric stimulation (26), and vertebral artery occlusion syndrome (21), and may be ascribed to short-term adaption of the vestibular imbalance.

## Limitations

There are several limitations in our study. First, we only quantified paroxysmal downbeat CPN, and did not include other types of paroxysmal CPN such as geotropic or apogeotropic horizontal nystagmus during head turning to either side while supine. However, our observation was also similar in apogeotropic CPN (Supplementary Video 3). Second, for the patient's safety, we set the maximum rotation velocity at 40 °/s during the motorized chair rotations. Thus, the characteristics of paroxysmal CPN during the head rotation at higher velocities remain to be determined. Third, due to severe vertigo, some patients involuntarily closed their eyes just after the positioning, and this might have biased the maximal SPV and TC. Finally, we couldn't measure the accurate acceleration and deceleration of the head motion during the SHH tests, so it was not possible to further elucidate the effects of velocity and acceleration on paroxysmal CPN.

## Data Availability Statement

The raw data supporting the conclusions of this article will be made available by the authors, without undue reservation.

## Ethics Statement

The studies involving human participants were reviewed and approved by Institutional Review Board of Seoul National University Bundang Hospital (IRB No. B-1908/558-103). Written informed consent to participate in this study was provided by the participants. Written informed consent was obtained from the individual(s) for the publication of any potentially identifiable images or data included in this article.

## Author Contributions

XL acquired and analyzed the data and wrote the manuscript. H-JK, J-HL, J-YC, and XY analyzed and interpreted the data and revised the manuscript. J-SK designed and conceptualized the study, interpreted the data, and revised the manuscript. All authors contributed to the article and approved the submitted version.

## Conflict of Interest

The authors declare that the research was conducted in the absence of any commercial or financial relationships that could be construed as a potential conflict of interest.

## Supplementary Material

Supplementary Video 1 Paroxysmal downbeat nystagmus induced during slow and fast manual straight head hanging in patient 21. With slow positioning, no nystagmus was induced, and the pre-existing spontaneous downbeat nystagmus was even suppressed. During fast positioning, in contrast, paroxysmal downbeat nystagmus was induced for several seconds.

Supplementary Video 2 Paroxysmal downbeat nystagmus induced during straight head hanging using a motorized rotation chair in patient 21. During rotation at a velocity of 10°/s, only small downbeat nystagmus was observed for a couple of seconds. During rotation at a velocity of 40°/s, in contrast, strong downbeat nystagmus was induced for about 7 s, which was followed by a small upbeat nystagmus.

Supplementary Video 3 Effect of positioning velocity on paroxysmal apogeotropic central positional nystagmus during head turning to either side while supine. During fast head rotation, strong paroxysmal apogeotropic nystagmus was induced with the maximum slow phase velocity (SPV) at 28.1°/s and the time constant of 3.7 s, and was followed by a persistent nystagmus (mean SPV:1.9°/s). In contrast, the induced paroxysmal nystagmus was absent or minimal after slow head rotation, but the persistent nystagmus was still observed at the mean SPV of 2.1°/s.
